# The risk of drug resistance during long-acting antimicrobial therapy

**DOI:** 10.1098/rspb.2022.1444

**Published:** 2022-11-09

**Authors:** Anjalika Nande, Alison L. Hill

**Affiliations:** ^1^ Program for Evolutionary Dynamics, Harvard University, Cambridge, MA 02138, USA; ^2^ Institute for Computational Medicine, Johns Hopkins University, Baltimore, MD 21218, USA

**Keywords:** drug resistance, long-acting therapy, mathematical modelling, microbial evolution, HIV, viral dynamics

## Abstract

The emergence of drug resistance during antimicrobial therapy is a major global health problem, especially for chronic infections like human immunodeficiency virus, hepatitis B and C, and tuberculosis. Sub-optimal adherence to long-term treatment is an important contributor to resistance risk. New long-acting drugs are being developed for weekly, monthly or less frequent dosing to improve adherence, but may lead to long-term exposure to intermediate drug levels. In this study, we analyse the effect of dosing frequency on the risk of resistance evolving during time-varying drug levels. We find that long-acting therapies can increase, decrease or have little effect on resistance, depending on the source (pre-existing or de novo) and degree of resistance, and rates of drug absorption and clearance. Long-acting therapies with rapid drug absorption, slow clearance and strong wild-type inhibition tend to reduce resistance caused by partially resistant strains in the early stages of treatment even if they do not improve adherence. However, if subpopulations of microbes persist and can reactivate during sub-optimal treatment, longer-acting therapies may substantially increase the resistance risk. Our results show that drug kinetics affect selection for resistance in a complicated manner, and that pathogen-specific models are needed to evaluate the benefits of new long-acting therapies.

## 1. Introduction

In recent decades, highly effective drugs have helped reduce morbidity and mortality of chronic viral infections, like those caused by the human immunodeficiency virus (HIV) [1] and hepatitis B (HBV) [2] and C (HCV) [3] viruses. However, drug resistance can evolve rapidly within individual hosts owing to the large population sizes and high replication and mutation rates of many viruses, rendering treatments ineffective [4]. Similar problems complicate treatment for chronic bacterial infections like tuberculosis (TB) [5]. The long treatment courses (months–years) required for chronic infections increase the opportunity for pathogens to adapt.

Effective treatments can also fail owing to non-adherence (missed doses). Typical rates of adherence for long-term medications are between 50% and 75% [6], which may be far less than what is needed for effective treatments. For some HIV antiretroviral therapies, studies have estimated that patients need near perfect (greater than 95%) adherence for viral suppression [7], while for HBV, adherence levels greater than 80% have been associated with an approximately 90% reduction in the rate of treatment failure [8].

Long-acting drugs are being developed to help address the problem of imperfect adherence [9]. For example, a two-drug injectable treatment regimen (cabotegravir/rilpivirine) was recently approved for treating HIV, and with a multi-week half-life it is administered only once every four or eight weeks as opposed to current daily dosing [10,11]. Large investments are being made in developing long-acting treatments for HCV and TB, and prophylaxis for TB and malaria [12,13], with some success already in animal models [14–16]. Long-acting lipoglycopeptide antibiotics are already available to treat bacterial skin infections [17]. Monoclonal antibodies are an emerging treatment for infectious diseases which can be engineered to have long half-lives [18]. While long-acting therapies are likely to increase overall patient adherence, their affect on resistance is unknown. Many studies have shown that sub-optimal adherence to daily pills contributes to resistance [19–24], but it is also possible that the long-term exposure to intermediate drug levels between doses of a long-acting drugs could facilitate the evolution of resistance [25,26]. The goal of this study is to examine the role of drug dosing kinetics on the risk of resistance.

Drug resistance can arise from two sources: mutants that exist prior to treatment initiation or those that are produced during treatment [27,28]. The relative contribution of these sources towards resistance is difficult to separate experimentally, but has been thoroughly investigated in a generalized model of intra-host viral dynamics for a completely resistant mutant in the presence of constant drug efficacy [29]. However, recent work on evolution in fluctuating environments [30] shows that the fate of mutants that are under time-dependent selection pressures cannot necessarily be predicted by the time-averaged selective effect alone; suggesting the effect of drug kinetics should be important. Numerous studies have integrated pharmacokinetics into mathematical models of infection dynamics [31–42], and have shown that the likelihood of generating and selecting for drug resistance depends on fluctuating drug levels. However, no studies have systematically studied the impact of changing a drug profile, as will occur with the reformulation of drugs into long-acting therapies.

In this study, we expand previous models to analyse the effect of drug kinetics on the evolution of resistance, focusing specifically on the frequency of drug dosing. We incorporate competition between wild-type (WT) and resistant strains, the fitness costs and benefits of resistance, pharmacologically relevant drug kinetics and treatment adherence. We consider pre-existing and rescue (de novo) mutations as well as mutations arising from reactivation of latent infection. This framework allows us to determine the conditions under which long-acting therapy promotes versus inhibits the development of drug resistance.

## 2. Model

**Figure 1.**
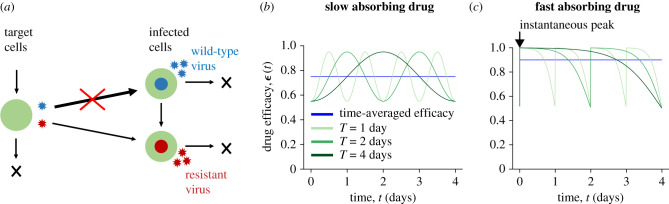
Model schematic and example time-dependent drug efficacy profiles. (*a*) Schematic of the viral dynamics model consisting of uninfected target cells (green) and cells infected with either the wild-type (WT) (blue) or drug-resistant virus (red). We assume treatment blocks infection with the WT (red cross), while the resistant strain can still (at least partially) infect cells. The resistant virus is assumed to have a fitness cost, so in the absence of treatment, the WT is more infectious and dominates the population. The resistant strain can be produced via mutation from the WT. Time-dependent drug efficacy *ε*(*t*) under the (*b*) slow and (*c*) fast absorbing drug models. Green curves correspond to drug profiles with the same time-averaged (blue line), maximum and minimum efficacy, but with different dosing periods, *T*.

To understand the impact of drug kinetics on the evolution of resistance, we used a stochastic model of viral dynamics within individual hosts [43] (see the electronic supplementary material, Methods for equations). This model (figure 1*a*) describes the interactions between target (uninfected) cells, drug-susceptible WT virus, and a drug-resistant virus strain, in the presence of treatment. While this model was designed for chronic viral infections, our results are generalizable to other infections with density-dependent growth and direct-acting, infection-blocking therapeutics (e.g. [38,39]). For now, we assume that the resistant mutant is generated from the WT by a single point mutation. We discuss the implications of more complex mutational pathways in the electronic supplementary material.

Like most infection models, pathogen fitness can be encapsulated by the basic reproductive ratio, *R*_0_, which is a composite of multiple individual parameters (electronic supplementary material, Methods). *R*_0_ is defined as the average number of new infected cells produced in a single replication cycle by one infected cell in an otherwise susceptible population [43]. A viral strain *i* can establish infection only if $R_{0}^{i}>1$. When there are multiple strains with $R_{0}^{i}>1$, competitive exclusion occurs and only the strain with highest *R*_0_ can sustain high-level infection. We assume that resistance is accompanied by some fitness cost, 0 ≤ *s* < 1. Consequently, in the absence of treatment, $R_{0}^{w}>R_{0}^{r}$, and infection is predominantly with the WT strain. The resistant strain is maintained in the population at low levels by a mutation-selection balance (electronic supplementary material, figure S1), which may lead to pre-existing resistance.

We assume that treatment reduces the infection rate by 1 − *ε*(*t*), where 0 < *ε*(*t*) < 1 is the time-varying drug efficacy. The drug is less efficacious against the resistant strain (*ε*_*r*_ < *ε*_*w*_). We are interested in a regime where an established WT infection is suppressed by treatment ($R_{0}^{w}>1$ and $R_{0}^{w}(1-\epsilon_{w})<1$), whereas the resistant strain is not ($R_{0}^{r}(1-\epsilon_{r})>1$). We consider two example drug efficacy models (electronic supplementary material, Methods): a ‘slow absorbing’ model in which drug absorption is gradual, such that the peak efficacy occurs in the middle of the dosing interval (figure 1*b*), and a ‘fast absorbing’ model in which drug efficacy peaks immediately after dose administration and then decays continuously until the next dose (figure 1*c*). This choice allows us to investigate the role played by the relative rates of drug absorption and clearance on the risk of resistance which is important for long-acting drugs as they are expected to have large differences in their absorption profiles [44]. With each of these models, we systematically varied the dosing interval while keeping the average efficacy the same (as well as the peak and trough efficacy). This is meant to mimic the scenario under which long-acting therapies are being developed: we assume that if the drug decay can be slowed down by some amount, the dosing interval is extended by the same amount.

Conceptually, there are two possible ways drug resistance can emerge to therapy [27,29]. Drug resistant strains may pre-exist at the start of therapy, since they are continually produced by the WT strain and exist at mutation-selection balance. Once the WT infection is suppressed by therapy, the resistant strain has less competition for target cells (the ‘resource’), and can potentially re-establish infection. However, establishment is not guaranteed: the population of pre-existing resistant mutants can be very small and subject to stochastic extinction. Resistant infection can also be established by mutants produced by residual WT replication during therapy (referred to as ‘rescue mutants’ following [29], a term borrowed from population genetics literature [45]), or later on in the treatment course owing to reactivation of latent infected cells (for certain infections). These mutants are also subject to stochastic extinction. The establishment probability of a resistant infection is, therefore, a result of complicated, time-varying birth–death dynamics and multiple model parameters (e.g. rate of target cell production, cost of resistance; see the electronic supplementary material for further details). In the following sections we analyse the effects of drug dosing intervals on this probability for different sources of mutants.

## 3. Results

### (a) Resistant mutants existing prior to treatment

**Figure 2.**
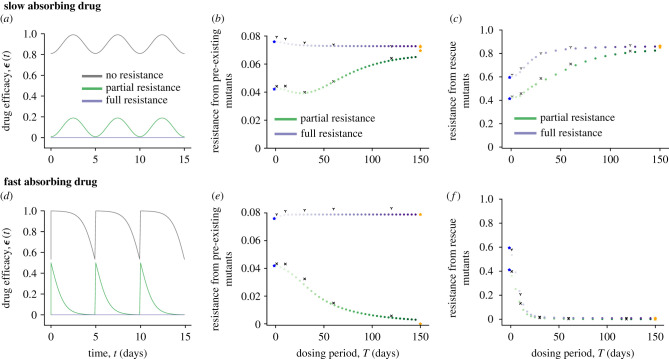
Effect of drug dosing intervals on the establishment of resistance owing to pre-existing and rescue mutants. Results from numerical simulations for the slow (top row) and fast (bottom row) absorbing drug models. (*a*,*d*) Drug efficacy *ε*(*t*) versus time for the WT and resistant strain. (*b*,*e*) Establishment probability for one pre-existing resistant mutant as a function of the dosing interval. (*c*,*f*) Probability that at least one rescue mutant is produced as a function of the dosing interval. Each dot represents the establishment or rescue probability in the presence of an oscillatory efficacy with a fixed period. Colours go from light to dark with increasing dosing intervals keeping constant time-averaged efficacy. Results are for mutants unaffected (purple) and affected (green) by the drug. Stars correspond to the probabilities for constant efficacies: time-averaged efficacy (blue) and efficacy at *t* = 0 (orange). The overlaid black marks are results from stochastic simulations of the model (electronic supplementary material, Methods).

We first examined the impact of the dosing interval on the evolution of resistance from pre-existing mutants, which depends both on their number at the time of treatment initiation and the subsequent establishment probability (*p*_est_) of each of them. Since only the latter depends on the treatment course, we calculated the establishment probability for a single pre-existing resistant mutant in the presence of treatment, from traditional daily therapy to long-acting multi-monthly intervals with the same average, maximum and minimum efficacy (electronic supplementary material, Methods). We first examined the case of a fully resistant mutant (a strain completely unaffected by the drug level; purple curves in figure 2*b*,*e*). For the slow absorbing drug profile, longer dosing intervals (i.e. long-acting therapy) always lead to lower probabilities of resistant mutants establishing. By contrast, for the fast absorbing drug profile, increasing the dosing period makes it more likely the resistant strain will establish. However in either case, the differences in *p*_est_ were minimal.

We next looked at the case where the mutant is only partially resistant (green curves in figure 2*b*,*e*). Overall the establishment probability was lower and more sensitive to the drug dosing frequency. When drug was absorbed more slowly between doses, longer dosing intervals only led to slightly lower establishment probability up to a certain maximal dose period (approx. monthly for the parameters we used), and then further increasing the period leads to even higher establishment probabilities than daily dosing. When drug was absorbed quickly, less frequent dosing decreased the establishment probability. Therefore, the effect of increasing dosing intervals on the risk of resistance owing to pre-existing mutants seems to depend strongly on both the degree of resistance and on the details of the drug kinetics curve.

We identified an important timescale in the system that helps explain the complex relationship observed between the dosing frequency and the risk of pre-existing resistance establishing. When therapy begins, the resistant strain has a selective advantage over the WT, but the establishment probability is extremely low until the WT strain is sufficiently suppressed to remove competition for the limited resource they share (i.e. target cells). Each individual mutant has a finite lifespan, and the lineage of this strain must survive this time period to make establishment likely.

We call this timescale the establishment time frame (ETF), and we discuss its derivation and dependence on model parameters in the electronic supplementary material, figure S2 and Methods. The effective drug levels during the ETF are the most relevant for resistance risk, explaining the observation of two limiting cases: when dosing is frequent, the drug undergoes many cycles during this time frame and the establishment probability is well-approximated by assuming constant efficacy at the time-averaged value (see blue stars in figure 2*b*,*e*). When drug dosing is very infrequent (i.e. long-acting therapy), the establishment probability approaches the value predicted from the earliest drug levels (orange stars in figure 2*b*,*e*).

Divergent results for partially versus fully resistant mutants can also be explained by selection forces acting during the ETF. Higher drug efficacy during this time period indirectly promotes the resistant strain by suppressing WT competition, but if the mutant is not completely resistant, the drug also causes some partial direct dose-dependent inhibition. Thus, for a fully resistant mutant, establishment is more likely for drug profiles with higher effective efficacy during the ETF (figure 2*b*,*e*), whereas for a partially resistant mutant the opposite is true when the inhibition by the drug is the dominant selection force (figure 2*b*,*e*). The trends described here are robust to the choice of parameter values, and we discuss this in detail in the electronic supplementary material, Discussion along with figures S3 and S4.

To summarize, when partial inhibition of the resistant strain during therapy is the dominant selection force, long-acting drugs tend to increase the risk of resistance owing to pre-existing mutants when the drug absorption is slow, and decrease the risk when drug absorption is fast. The converse is true when the degree of resistance is high and competition with the WT dominates instead.

### (b) Resistant mutants produced during treatment

Next, we examined the effect of dosing frequency on the evolution of resistance owing to de novo mutants produced during treatment. To contribute to treatment failure, a resistant lineage must first be produced by mutation during residual WT replication despite therapy and then have a strong enough selective advantage to escape stochastic extinction. We computed the probability that at least one such ‘rescue mutant’ is produced during treatment (electronic supplementary material, Methods), and analysed how it was affected by varying the frequency of drug dosing while keeping the average, maximum, and minimum drug efficacy fixed (figure 2*c*,*f*).

We found that for slow drug absorption, long-acting drugs tend to increase the risk of resistance (figure 2*c*). By contrast, for fast drug absorption, longer acting drugs reduce the risk of resistance (figure 2*f*). These trends are independent of the degree of resistance of the mutant strain, and are robust to variations in other parameters (see the electronic supplementary material, Discussion and figures S5 and S6 for details).

The ETF also plays an important role in explaining the risks of de novo resistance emerging, but through different mechanisms. The rate at which rescue mutants are produced depends on the product of the rate of mutant production from residual WT replication before infection is controlled, which is increased for lower drug efficacy during the ETF, and the establishment probability of these newly generated mutants, which may be increased for either higher or lower drug efficacy depending upon the degree of resistance of the mutant strain (as was seen in the case of pre-existence).

Our findings suggest that the impact of drug kinetics on the rate of mutant production dominates the effect on mutant selection (and establishment probability). Drug profiles with higher efficacy shortly after treatment begins (e.g. frequently dosed slow-absorbing drugs or long-acting fast absorbing drugs) are associated with lower risks of resistance from rescue mutants. There are two limiting cases again: when dosing is frequent, the rescue probability depends upon the time-averaged drug efficacy, whereas in the limit of very infrequent dosing it depends upon the initial drug efficacy.

In summary, long-acting therapy increases the risk of resistance owing to rescue mutations when drug absorption is slow, and decreases it when drug absorption is fast.

### (c) Resistant mutants produced owing to latency reactivation

The infection model considered so far is applicable to infections like HCV that do not have persistent infected cell populations [46]. As a result, the infection tends to be cleared quickly as long as resistance does not emerge, which is why the early drug kinetics play an outsized role in the risk of resistance. Persistence, however, is a characteristic of many chronic infections (e.g. HIV, HBV, TB, some types of malaria) [5,47] that makes it difficult to design effective treatment strategies to completely clear the infection. It is also an important source of resistance. Reactivation events from this pool (‘latent reservoir’) of persistent infected cells can lead to the introduction of resistant mutants that establish infection [35,48,49]. The rate at which resistant mutants are produced and go on to establish infection (‘rescue’ owing to reactivation) is dependent upon the details of the drug kinetics and hence, we expect it to be influenced by the drug dosing frequency. With this in mind, we investigated how long-acting therapy affects the emergence of a resistant infection owing to the reactivation of latent infected cells.

**Figure 3.**
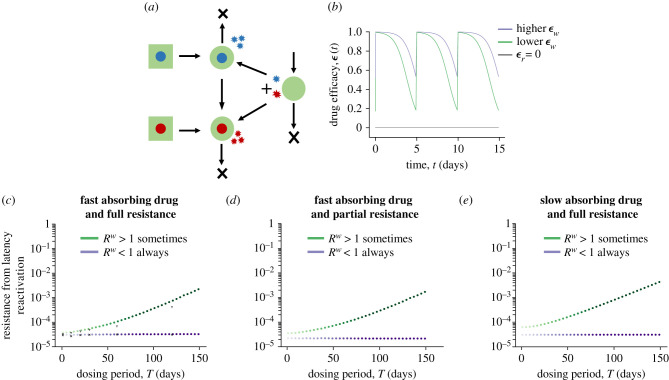
Effect of drug dosing intervals on the establishment of resistance owing to latency reactivation. (*a*) Model schematic showing the two ways in which resistant mutants can be produced via a latent infection (square). (*b*) Example drug efficacy *ε*(*t*) versus time curves for WT (*ε*_*w*_) and resistant (*ε*_*r*_) strains under the fast absorbing drug model. The purple curve corresponds to a high drug efficacy with *R*^*w*^ < 1 for all points of the drug cycle. *R*^*w*^ > 1 for approximately 23% of each period for the lower efficacy drug (green). (*c*–*e*) Average probability of rescue per latency reactivation event as a function of the drug dosing interval for different drug efficacy profiles and degrees of resistance. Colours darken with increasing *T* keeping constant time-averaged efficacy. Black marks in (*c*) denote results of the stochastic simulation of the model. See the electronic supplementary material, Methods for details on the parameter values.

We modelled two ways in which resistant mutants can be introduced during a persistent infection (electronic supplementary material, Methods)—reactivation of a latently infected lineage that is already resistant (we assume that a fraction of the latent population given by mutation-selection balance was resistant at the time it was seeded), or reactivation and subsequent replication of WT infection that generates a resistant mutant (figure 3*a*). Summing up these possibilities, we calculated the average probability of rescue per latency reactivation event (electronic supplementary material, Methods).

We found that depending upon the degree of WT inhibition between doses, long-acting therapies are associated with the same or increased risk of resistance as compared to frequent dosing (figure 3*c*). When the drug efficacy is high enough that the WT is suppressed (*R*^*w*^ < 1) for the entire the drug cycle, the average rate of rescue has no dependence upon the dosing interval (purple curve in figure 3*c*). In the absence of WT replication, resistance can arise only via mutants that pre-exist in the latent reservoir. Since we assume the rate of reactivation is constant and independent of the drug kinetics, the risk of resistance in this case does not depend upon the drug dosing frequency. However, when the drug efficacy is lower and the WT can replicate during the times of lowest drug levels (*R*^*w*^ > 1), despite still being suppressed overall (〈*R*^*w*^〉 < 1), the rate of rescue has a strong dependence upon the dosing intervals and increases for longer acting drugs (green curve in figure 3*c*). The slow decay of longer acting drugs leads to more time in the non-suppressive part of the dosing period, enabling the WT to undergo multiple rounds of replication before eventually getting suppressed by the next dose, and increases the chance that a resistant mutant is produced and establishes infection. These results are irrespective of the degree of resistance of the mutant strain (figure 3*d*) and the drug absorption rates (figure 3*e*).

In summary, during a persistent infection, long-acting therapies are associated with an increased risk of resistance if there is sub-optimal WT suppression in the lowest parts of the drug cycle.

### (d) Impact of non-adherence on resistance for different dosing intervals

**Figure 4.**
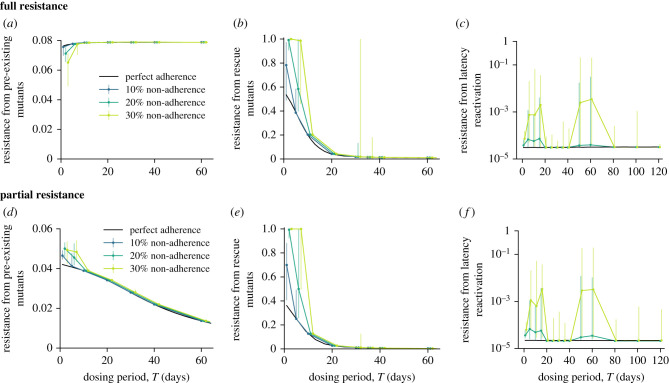
Effect of non-adherence on the establishment of resistance for different drug dosing intervals. Results for a mutant fully (top row) and partially (bottom row) resistant to treatment. (*a*,*d*) Establishment probability of pre-existing mutants. (*b*,*e*) Probability that at least one rescue mutant is produced. (*c*,*f*) Average probability of rescue per latency reactivation event. Results are for the fast absorbing drug model with the solid black line in each subplot corresponding to results for perfect adherence. Imperfect adherence results are medians of 100 iterations for pre-existence and rescue, and 500 iterations for latency. The error bars correspond to the interquartile range. *X*-axis positions are offset for ease of visualization.

The previous sections show how drug kinetics influence the risk of resistance when there is perfect adherence to treatment. In reality, treatment adherence is known to be sub-optimal, especially for chronic infections, and is associated with the risk of resistance for daily therapies. We, therefore, evaluated how imperfect adherence to therapy influences the relationship between drug dosing intervals and the risk of resistance. We assumed each scheduled dose was taken or missed randomly and independently with a probability given by the adherence level (electronic supplementary material, Methods) and analysed the effect of dosing intervals on the probability of a resistant infection owing to the different sources of mutations, for varying levels of adherence (figure 4). While in reality low enough adherence levels can allow for treatment failure to occur without resistance, just due to rebound of the WT strain, we were not interested in this effect and so ensured that the system stayed in the regime where the WT has a lower fitness than the resistant strain and is suppressed by treatment.

We found that for both pre-existing and rescue mutants, long-acting drugs were more robust to the effects of treatment non-adherence, with the same observed overall trends relating the risk of resistance to the frequency of dosing as for perfect adherence (figure 4*a*,*b*,*d*,*e*). Long-acting drugs are more robust in this case because the risk of resistance is mainly dependent upon the treatment strength during the ETF. The number of doses taken during this time frame decreases as the dosing interval increases, reducing the chance one of them is missed. As by definition the first dose is always taken, the risk of resistance is the same as that for perfect adherence when the dosing interval is equal to or larger than the ETF. These results are independent of the specific model chosen for adherence (see the electronic supplementary material, Methods and figure S7 for results with an alternative adherence model in which missed doses can be taken on any subsequent day, instead of waiting for the next scheduled dose).

In agreement with previous modelling work [35,39], we found that non-adherence generally—but not always—promotes the emergence of resistance. In the case of fully resistant pre-existing mutants, lower adherence actually lowers the establishment probability (figure 4*a*). With lower adherence, the WT infection is less effectively suppressed, which increases competition for target cells and makes it more difficult for the resistant strain to establish. This highlights the more general observation from other modelling studies [27,38,50] and experiments [51,52] that an ‘aggressive’ treatment (e.g. high initial drug levels) does not necessarily lead to reduced resistance risk.

Depending upon the drug dosing frequency, imperfect adherence can significantly increase (by orders of magnitude) the risk of resistance during a persistent infection irrespective of the degree of resistance of the mutant strain (figure 4*c*,*f*). The non-monotonic pattern of resistance risk versus dosing period and large error bars imply that the resistance risks are very sensitive to the state of the system at the time the mutant arises. When drugs are very long-acting (drug dosing period larger than approximately two months), imperfect adherence does not play much of a role—missing a dose causes WT infection to rebound without selecting for resistance. However, for shorter dosing periods the risk of resistance is predominantly increased, apart from an intermediate regime. This trend is a combined effect of the rate at which resistant mutants are produced from the WT and competition between the two strains for uninfected cells once the resistant mutants have been produced. The risk of resistance is highest in the regime where missing a dose leads to a burst of WT replication and mutant production, but where there is enough WT suppression overall that a resistant strain can establish infection. This is also why the overall rates of resistance are higher if we instead consider an alternative adherence model where individuals are given the chance to take a missed dose on any subsequent day, instead of waiting for the next scheduled dose (electronic supplementary material, Methods, Discussion and figure S7). Although on average the number of missed doses are equivalent in both models, the distribution of the number of days between doses is narrower in the second model and results in drug levels reaching highest-risk intermediate levels more frequently.

The results presented in this work correspond to a model where we assume that the resistant strain is one mutational step away from the WT. However, we find that the results also qualitatively hold for a more complex mutational pathway (see the electronic supplementary material, Methods and figure S8 for more details).

To summarize, longer acting drugs are more robust to the effects of imperfect adherence when drug resistance is expected to arise owing to mutants pre-existing or generated early on after the start of treatment. During a persistent infection, non-adherence to long-acting drugs can dramatically increase the risk of resistance occurring if latent infection reactivates compared to daily dosing.

## 4. Discussion

New long-acting antimicrobial therapies are being developed to improve patient adherence and reduce treatment failure [9]. Although these drug formulations are likely to reduce missed doses, it is unclear whether this alone will lead to better treatment outcomes. Therapies with extended half-lives can lead to long-term exposure to intermediate drug levels, which could promote the evolution of drug resistance [25,26]. A systematic study of the relationship between drug dosing kinetics and the risk of resistance is, therefore, essential to the development and optimization of new long-acting therapies.

In this study, we found that the interplay between time-varying drug concentrations in the body and competing pathogen strains results in a complicated effect of drug dosing intervals on the evolution of resistance (table 1). Using models of infection dynamics within hosts, we show that when resistance mutations pre-exist before treatment and confer complete resistance, long-acting therapies increase the resistance risk if drug absorption is fast and reduce it if drug absorption is slow. These trends can be reversed if pre-existing mutants confer only partial resistance. These opposing results suggest that in cases where complex resistance pathways exist, full disease and treatment-specific models that attempt to account for realistic fitness landscapes of resistance—such as the model developed by Raja *et al.* [53] or the one developed by Kirtane *et al.* [54]—will be needed to evaluate the risks of resistance and guide treatments. When the main source of resistance is mutants produced de novo via residual WT infection after treatment begins (‘rescue’), then irrespective of the degree of resistance, long-acting drugs reduce the resistance risk if drug absorption is fast and increase it if drug absorption is slow. Our results demonstrate that resistance risk owing to both pre-existing and rescue mutations is dominated by drug kinetics (treatment strength) and infection dynamics in a short initial time window after treatment begins, which we call the ‘establishment time frame’. It is worth noting that our analysis essentially amounts to calculating the fixation probability of a mutant under time-varying selection pressures, a complicated problem that has been studied in the field of population genetics. We relate our findings to previous work in the electronic supplementary material.

**Table 1.** Summary of the effect of long-acting drugs on the risk of resistance during therapy. (‘↑’ means that the probability of drug resistance emerging during long-acting therapy is higher compared to daily dosing (with the same average, peak, and minimum levels), and ‘↓’ means the opposite. Dotted arrows indicate minimal effect. For resistance arising from mutations that are either ‘pre-existing’ at the time treatment starts or from ‘rescue’ (de novo) mutations produced by residual replication before WT infection is suppressed, the speed of drug absorption (relative to clearance) is the important pharmacokinetic factor determining resistance risk. For resistance arising from reactivation of persistent infection long after therapy initiation, the inhibitory effect of the minimum drug level is the most important pharmacokinetic factor determining resistance risk (with a more minor role played by the drug absorption rate and degree of resistance). Results come from simulations with a specific infection model and parameter values, and while our analyses suggest trends are relatively general, the magnitude of the effect will change with model details (see the electronic supplementary material, Discussion for more details).)

		degree of resistance	
source of resistance	drug kinetics model	full	partial
pre-existing	slow drug absorption	⇣	↑ ⇣
	fast drug absorption	⇡	↓ ⇡
rescue	slow drug absorption	↑	↑
	fast drug absorption	↓	↓
latency reactivation	suppressive minimum drug level	no change	no change
	non-suppressive minimum drug level	↑	↑

Treatment for infections such as HIV and TB can be compromised by persistent sources of infection which are not rapidly cleared by therapy. For resistance arising from reactivation of persistent reservoirs, early drug kinetics (and thus the absorption speed of long-acting therapies and the degree of resistance) become less important. Resistant mutants can arise at any time from pre-existing mutant populations in the persistor pool, or get generated during breakthrough replication of WT cells from the persistent population when drug levels are low. We found that during persistent infection, longer-acting therapies increase the risk of resistance if the drug is not fully suppressive during the lowest drug levels. Long-term exposure to low drug levels can allow WT populations that reactivate from latency to temporarily grow, significantly increasing the chance that resistant mutants are generated and expand. Individual physiological differences in drug absorption, distribution, metabolism and excretion mean that the suppressive action of therapy can vary significantly across a population, and previous studies have stressed the need to individualize treatment regimens [32,55]. Our findings suggest that this need might be even more acute for longer-acting therapies.

Imperfect treatment adherence is a major challenge for long-term antimicrobial therapy and can facilitate the emergence of resistance. While the development of long-acting therapies was motivated by the need to improve adherence, their robustness to missed doses has not been evaluated. Mathematical models have helped provide valuable insights into understanding the complex and at times surprising effects of treatment non-adherence [31–36,39,56]. Extending this work to long-acting therapies, we find that the effects of imperfect adherence depend on whether there is a persistent source of infection. In its absence, long-acting drugs are more robust to the effects of imperfect adherence compared to more frequent dosing, since it is less likely doses will be missed in the critical early time frame during which WT infection is cleared before resistant strains can be produced or effectively compete for resources. However, if there is a persistent population from which infection can reactivate during later periods of non-adherence, then some intermediate dosing frequencies lead to dramatically higher resistance risks.

Our model assumes a single well-mixed pathogen population. In reality, pathogen subpopulations can reside in tissues and organs where drug absorption and pathogen dynamics may be significantly altered [57–59]. Previous work has considered the consequences of spatial heterogeneity on treatment efficacy and the role it can play in facilitating the emergence of drug resistance [60–66]. In our analysis, the rate of drug absorption played an important role in determining the risk of resistance to long-acting therapies. Since drug absorption and clearance can differ between tissues, we suspect that the relationship between the frequency of dosing and the emergence of resistance could be more complex for compartmentalized infections.

The injectable combination cabotegravir/rilpivirine is the only long-acting antiretroviral therapy for HIV in late-stage of clinical trials, and after showing non-inferiority to the current standard daily oral dosing it was recently approved for use in many countries [10,11,67,68]. Our analysis provides some insight into what to expect long-term. In these trials, participants initiating therapy were given standard daily doses until viral suppression was achieved, after which they were randomly assigned to a longer dosing schedule (four or eight weeks) or kept at daily dosing as a control. Our analysis suggests that by switching to longer-acting therapy only after viral suppression is achieved, the risk of resistance from pre-existing or rescue mutations during the critical ETF may be negated. HIV infection will still persist indefinitely in the latent reservoir and may reactivate during periods of non-adherence, but results suggest that long-acting therapy will not be associated with an increased risk of resistance if drug levels are kept high enough in all individuals to ensure WT suppression even at the lowest points of the drug cycle, which existing pharmacokinetic data supports [69,70]. The rates of non-adherence for these monthly or bi-monthly injections has yet to be determined.

Long-acting drugs are also being considered for prophylactic use to improve adherence and increase effectiveness in preventing infection, for example for HIV and malaria [14,71,72]. For long-acting preventative therapy, there are concerns about the emergence of resistance owing to long-term exposure of intermediate drug levels if prophylaxis is insufficient to prevent infection, or is initiated during an undiagnosed acute infection [73,74]. Penrose *et al.* [74] report an instance of infection with WT HIV and subsequent selection of a resistant virus owing to persistent exposure to long-acting pre-exposure prophylaxis (PrEP). In addition, Radzio-Basu *et al.* [73] show in a macaque model that initiating long-acting PrEP during acute simian-human immunodeficiency virus infection can frequently select mutations conferring resistance to therapy and are maintained for several months. Although we have not considered prophylactic use directly, some of our results seem qualitatively applicable. The risk of resistance if prophylaxis is unknowingly started during acute infection may be similar to our findings for the risk due to rescue mutations after therapy initiation. The risk of resistance during breakthrough infections is likely to depend on drug kinetics in a similar way to that of reactivating persistent infection, but the chance of breakthrough infection itself may require a more specific model of transmission.

Our findings underscore the importance of explicitly modelling drug kinetics, since time-averaged drug efficacy is a poor proxy for resistance risk. Future work should focus on developing application-specific models that use data-driven pharmacodynamic, pharmacokinetic and evolutionary parameters. Such models can help guide future dose-optimization and implementation tasks for long-acting therapies for globally important infectious diseases.

## Data Availability

Our code is open access on GitHub: https://github.com/anjalika-nande/drugkinetics-resistance. The data are provided in the electronic supplementary material [75].
